# Supplementary material for: Are carbon emissions trading and green financial instruments synergistic? -Comprehensive quantitative research based on content analysis
Source: PLoS One. 2024 Mar 7;19(3):e0298601. doi: 10.1371/journal.pone.0298601 (PMC10919601; doi:10.1371/journal.pone.0298601)
Supplement: S1 Data — (ZIP) [file pone.0298601.s001.zip › Data/地级市Z_F工作报告.zip!/╕ú╜¿/╚2├≈╩╨/└┤╫╘╣1⁄2╓┌║┼í╛┬φ┐╦╩2╛▌═°í┐_2006.docx]

全面实施“十一五”规划，为三明在海峡西岸经济区建设中加快崛起而努力奋斗 顺势而为在融入海峡西岸建设中取得成效

　　2005年，我们在省委、省政府和市委的正确领导下，高举邓小平理论和“三个代表”重要思想伟大旗帜，全面贯彻党的十六届三中、四中、五中全会精神，树立和落实科学发展观，以开展保持共产党员先进性教育活动为动力，主动融入海峡西岸经济区建设，全力推进经济持续较快增长，加快构建社会主义和谐社会。经过全市人民的共同努力，全面完成和超额完成市十届人大三次会议确定的各项目标任务。初步统计，2005年全市生产总值392.1亿元，增长10.5%；全社会固定资产投资167.85亿元，增长29.7%；地方级财政一般预算收入19.33亿元，同口径增长14.2%；实际利用外商直接投资1.42亿美元，增长56%；外贸出口4.6亿美元，增长85.1%；社会消费品零售总额110.1亿元，增长11.7%；城镇居民人均可支配收入11397元，增长10%；农民人均纯收入4209元，增长8.3%；居民消费价格总水平102.2%;城镇登记失业率5.47%,回落0.25个百分点；人口自然增长率5.8‰。一些重要领域、重点项目取得新突破：

　　———全国唯一的海峡两岸（三明）现代林业合作实验区获准设立。成功举办首届海峡两岸(三明)林业合作暨投资贸易洽谈会,6个功能区建设全面展开。

　　———交通基础设施建设实现突破。第一条快速出省通道福（州）银（川）高速公路三明段建成通车，泉（州）三（明）高速公路全线动工，三明机场开工续建，向（塘）莆（田）铁路线路走向基本确定，农村公路改造、硬化1450公里，居全省第一位。

　　———一批生产性大项目取得重大进展。三钢钢产量突破300万吨,将乐红狮年产400万吨水泥一期工程等项目竣工投产,三钢80万吨中厚板、青纸20万吨高强瓦楞纸等项目动工。

　　———特色农业加快发展。竹业产值增幅居全省第一位；中国红豆杉产业可持续发展国际论坛在我市召开，人工种植红豆杉深加工产值、利税分别增长42.9%、59.6%；花卉苗木取得2008年奥运工程订单，实现批量出口日本、韩国；大灾之年烟叶产量仍居全国前列，质量继续领先；肉牛、肉羊产业化发展态势良好；120个产品获无公害农产品、绿色食品、有机食品认证。

　　———外贸出口快速增长，项目对接亮点凸显。外贸出口当年新增2.12亿美元，增幅全省第一。参加第三届项目成果交易会，对接合同项目数和投资额分别居全省第一、第二位。

　　———成功创建一批品牌。第三次荣获全国创建文明城市工作先进城市称号，首次进入全国社会治安综合治理优秀地市行列。沙县被评为全国文化先进县，泰宁当选中国十佳魅力名镇。我市水稻育种制种专家刘文炳研究员培育的超级稻Ⅱ优28号，创水稻单产世界纪录。

　　———中心城市加快拓展。市区、沙县一体化和三明、永安城市联盟启动，立丰印染、厦工三重搬迁改造迈出实质性步伐，三明至沙县道路照明工程建成，荆东、小蕉工业园区加快开发，市区环境综合整治取得实效。

　　———教育事业成效明显。高考成绩继续位居全省前列，三明职业技术学院正式成立，三明学院列入省级科技计划项目数居全省新建本科院校首位。

　　———惠民政策得到落实。全面免征农业税及其附加，取消除烟叶以外的农业特产税，全市农民受益8000多万元。累计兑付种粮农民直补款和良种补贴711.59万元，受益农民41.87万人。“两免一补”政策、高校贫困学生助学工作落实，2.58万名困难学生得到资助。实施政府津贴制度，农民技术员、乡村医生和计划生育管理员队伍建设加强。农村劳动力转移培训人数居全省第一位。1901个困难乡镇、村“两委”和城市社区居委会运转经费得到补助。拨付各类应急救灾和灾后重建资金7316万元，重建家园的受灾户春节前全部迁入新居。

　　2005年目标的顺利实现，标志着历经“十五”发展，三明改革开放和现代化建设进入新阶段。

　　（一）综合实力明显增强。“十五”期间，全市生产总值保持全省第五位,年均增长9.5%。2005年财政总收入38.91亿元,五年翻了一番。全社会固定资产投资累计突破500亿元,超过1952—2000年的投资总和。外贸进出口累计13.85亿美元，比“九五”期间翻了一番；实际利用外商直接投资累计突破4亿美元。社会消费品零售总额年均增长9.6%。城镇居民人均可支配收入、农民人均纯收入年均分别增长8.9%、5.7%。2005年金融机构各项存款余额345.56亿元，五年翻了一番；各项贷款余额235.43亿元，比2000年增长79%。居民消费价格总水平年均上升1.4%，城镇登记失业率年均5.65%，人口出生率年均11.3‰。

　　（二）经济结构不断优化。2005年三次产业结构比例为24：39：37，第二产业比重比2000年提高7个百分点。五年累计实施市级重点项目193项，其中生产性项目112项，64项已投产。农业综合生产能力提高，粮食产量稳定在126万吨以上，12家企业进入国家、省级农业产业化龙头企业行列，特色经济规模不断壮大。2005年规模以上工业总产值比2000年增长1.3倍，工业经济效益逐步提高；“3+1”产业集群加快发展，2005年实现产值占规模以上工业产值的53.4%。各类工业园区（集中区）加快建设，正在成为重要的经济增长点。运输、商贸、通信、中介等服务业持续增长，金融保险业运行良好，泰宁国家地质公园跻身世界地质公园行列。县域经济发展活力增强，综合实力提升。非公经济迅速崛起，产值、出口、上缴税收五年实现翻番。

　　（三）基础设施持续突破。高速公路通车里程218公里，国道、省道改造467.9公里，72.4%的行政村通水泥路，永（安）武（平）、永（安）宁（化）高速公路等重大交通项目前期工作进展顺利。街面电站等重大能源项目建设扎实推进，电力总装机容量达到207万千瓦，五年增加53万千瓦。农村水利等基础设施建设持续推进，气象、水文工作不断加强，防灾救灾减灾体系日渐完善。信息化基础设施建设加快，市县两级政务网建成，全市行政村实现村村通电话。2005年城镇化水平达到42.1%，比2000年提高3.5个百分点，市区、沙县一体化概念性规划完成，县城、县域次中心和村镇建设取得新进展。

　　乘势而上在加快海峡西岸建设中扎实作为

　　（一）加强“三农”工作，促进农民增收

　　以工业化带动农业产业化。落实粮食生产、流通、储备、安全责任，提高粮食综合生产能力。以龙头企业引领发展，以品牌壮大规模，巩固发展水果、茶叶、畜牧、水产、菌业、蔬菜、建莲等传统产业，加快发展优质烟叶、笋竹、肉牛、肉羊等特色产业，大力发展红豆杉、花卉苗木等新兴产业，壮大县域特色经济。逐步推进农业标准化建设，完善农产品质量认证和质量安全准入制度，打造产业、产品名市、名县，争创一批无公害农产品、绿色食品、有机食品。

　　千方百计增加农民收入。提高农民发展种养业收入，增加农民从事二、三产业收入，建立健全减轻农民负担长效机制。加大扶贫开发力度，通过产业扶贫等办法，促进低收入农户脱贫致富。实施农村劳动力转移培训,做好劳务派遣工作,新增转移农村劳动力3万人。鼓励、扶持发展各类农民合作经济组织，壮大农民营销队伍，搞活农产品流通。

　　加快农业科技进步。深化粮钱丰收竞赛活动，健全良种繁育体系，实施“科技入户”工程，创建科技示范乡镇，加快现代农业示范园区建设，促进农业科技成果产业化。实施“一县一业”星火计划，推进沙溪沿岸星火技术产业带建设，抓好一批星火密集区、星火产业基地和星火创新龙头企业。完善农业155服务网络，进一步深化“村会协作”，加强高致病性禽流感等重大疫情的防控，做好农、林、水产病虫害防治工作。

　　落实支农惠农政策。抓紧制定新农村建设总体规划和实施方案，统筹谋划新农村建设的目标、任务、步骤和项目。实施一批国家农业综合开发、土地整理、烟田基础设施等项目建设，推进“六千”水利工程建设，加快修复水毁水利工程。继续推动农村公路建设，完成水泥路面改造、硬化700公里。实施农村户用沼气工程建设，加快推进农村饮水、电网、通信等基础设施建设，继续增加农村教育、文化、卫生等方面的投入。充分发挥农技员、国土资源和规划建设协管员、计划生育管理员、乡村医生和治安协调员的作用，更好地服务农业、农村和农民。

　　（二）推进自主创新，加快产业集聚

　　全力突破工业。靠龙头企业集聚合力，靠项目组团延伸产业链，靠产业集群激活存量、扩张增量。冶金及压延产业对接浙江永康等五金产业发达地区，围绕80万吨中厚板、30万吨冷轧钢板等项目，增加产品加工环节；林产工业建设年产45万立方米刨花板、20万吨高强瓦楞纸等项目，引进广东、浙江、江苏等地的品牌企业，联手开发家具、包装等关联行业，拉长产业链；机械与汽车零配件产业主攻中重型载重汽车、工程机械、橡塑机械等项目，做大产业规模；生物医药产业主攻原料药，抓好红豆杉提纯紫杉醇、废次烟叶提取茄尼醇等项目，推进植物制药等快速发展。抓好宁化钨矿、尤溪和大田铅锌矿等矿产品开发，加快发展永安、将乐新型干法旋窑水泥，建设一批纺织、化工和绿色食品加工、新型电子元器件等大项目。对重点乡镇、重点企业实施重点扶持、重点服务,抓好30个工业重镇，壮大60家骨干企业。

　　加快转变经济增长方式。靠自主创新改造提升传统产业，抓好100个投资千万元以上工业项目，特别是15个在建亿元以上项目，抓紧10个亿元以上项目前期工作，抓好一批新技术、新产品攻关。靠品牌调整结构，重点支持“铙山”高档薄型包装纸等争创国家级品牌，新创一批省级品牌。靠园区集聚要素、集约发展，重点建设8个省级工业园和一批专业工业集中区，提高项目供地率、落地率和土地利用集约率。靠发展循环经济节约成本、提高效益，重点建设列入国家、省里的59个循环经济项目，抓好31家清洁生产试点企业。

　　整合提升服务业。引进厦门商业集团、永辉等知名企业，重组商贸龙头企业，改造提升三明综合市场等传统市场，建设科技会展中心等一批重点项目，举办系列会展活动，整合做大一批第三方物流企业，策划、开发大型物流中心和农业综合服务中心。拓宽消费领域，培育消费热点，大力实施农村市场建设，新增一批面向农村的商品配送中心和“农家店”。以泰宁世界地质公园为龙头，联动推出精品线路，联手拓展市场,壮大旅游企业,力争游客量突破500万人次，旅游收入增长20%以上。

　　（三）强化项目带动，扩大投资需求

　　抓紧抓实重点建设。围绕项目抓具体、抓系列、抓持续、抓趋势，继续提高我市项目占全省重点项目盘子的比重，提高重点建设投资占固定资产投资的比重，提高工业项目投资占重点建设投资的比重。列入今年省级重点项目42个，其中跨地市项目7个，年度投资55亿元，增长10.2%；安排市级重点项目90个，年度投资80亿元，增长11%。

　　加快基础设施建设。抓紧泉三高速公路施工，启动向莆铁路、永武高速公路控制性工程，完成永宁高速公路“预可”审批和“工可”报告编制，深化永（安）长（汀）铁路前期工作，编制宁德至邵武延伸到建宁、沙县经大田至厦门、永安至漳州高速公路路线规划方案，推进国道、省道“改善工程”，抓好三明机场建设，推进沙溪河二期航运工程。加快电力建设和电网改造，推进街面电站等项目建设，力争永安火电厂扩建工程获国家发改委核准，开工500千伏三阳变和220千伏金沙变等主干电网项目，实施城市、县城电网建设与改造工程。

　　（四）创新体制机制，增强发展活力

　　深化各项改革。继续推进工贸企业改革，抓好厦工三重、双轮化机、三明机床3家企业异地扩改，加快建筑业企业改革与发展，稳妥推进上市公司股权分置改革。建立和完善国有资产监督管理制度，维护国有资产权益。全面推进农村综合改革，深化农村税费改革，巩固、深化集体林权制度及其配套改革，稳步实施乡镇机构、农村义务教育体制等改革。探索跨县域利益共享机制，以林产工业为试点，以满足龙头企业的资源需求为重点，跨县域集中资源，跨县域配置要素，跨县域分享税收。

　　提升非公有制经济。落实省、市扶持非公有制经济发展的政策、措施，拓展投资领域，突出抓好一批投资千万元以上的项目。引导私营企业提高素质，向龙头企业靠拢，向工业园区（集中区）集聚，向高新技术产业延伸，重点培育铙山纸业、健盛食品、三和食品、高科橡胶、宝华林纺织等一批行业“单项冠军”。发展壮大商会经济，着力抓好28个重点商会投资项目。

　　加强财税金融工作。大力培植财源，明确财政资金扶持导向，主要用于重点产业、重点企业扩大再生产和自主创新。坚持依法治税，强化税收征管，促进财政收入稳定增长，逐步解决县、乡财政困难。调整财政支出结构，重点保障政权建设、公共事业发展和社会保障等支出。稳妥推进国库集中支付改革，深化部门预算改革，规范政府采购行为，防范和化解政府债务风险，加强财政资金运行监督。健全政银企会商制度，改善金融服务，增加信贷投入，扩大异地贷款，引导民间投资。完善信用担保体系，支持、促进担保机构做大做强，为中小企业发展提供融资服务。推进农村信用社改革，进一步改善金融生态，防范金融风险。

　　（五）拓展对外经贸，提高开放水平

　　继续实施外经贸追赶战略。保持出口政策的连续性，支持外贸企业做大做强，鼓励大宗地产品出口，提高加工贸易出口比重，拓展新兴出口市场。抓好招商引资，推出高速公路市级股权出让等项目，吸引战略性合作伙伴，推进重大基础设施项目外资化、民营化。积极组织参加“6·18”项目成果交易会、“9·8”投洽会等大型经贸活动，开展“3+1”产业集群招商和工业、农业、社会事业等专场招商，扩大利用外资规模与实效。

　　推进明台交流与区域经济协作。举办第二届海峡两岸（三明）林业博览会暨投资三明推介会，引进有实力的台商，设立台资项目集中区，支持办好现有台资企业，深化大金湖与日月潭对接，提升明台合作。推进与厦门、泉州等地的山海协作，加强与闽东北、闽西南以及闽赣周边地区的区域协作，联手推动跨市、跨省项目建设。做好侨务工作，扩大明港澳交流与合作。

　　（六）壮大中心城市，带动城镇建设

　　推进市区“南拓北扩西进”。抓紧荆东、小蕉工业园区的开发和项目引进，加快贵溪洋、台江、东霞等片区的规划、建设，有序搞好土地收储与供应，抓紧建设阳光嘉园、龙岗小区等商品房和省安小区等经济适用房。续建三明沙溪河大桥等项目，建设西江滨南路二期、白沙路等项目，改造东新五路等路网，抓好城市园林绿化建设，实施社区基础设施改造，增强城市配套功能，改善市区人居环境。推进城市管理体制改革，开展市容市貌专项整治，提高城市管理水平。

　　加快永安—市区—沙县繁荣带建设。完成三明市城市总体规划修编，策划融机场、铁路、高速公路为一体的综合交通枢纽，开通市区到沙县公交线路，启动市区至沙县滨河旅游项目建设，规划物流园区，共建一批基础设施、产业发展等项目。抓好三明—永安工业走廊和莘口、贡川连接点规划建设，实施一批重要文体设施等项目，推动城市联盟取得进展。

　　抓好县城和小城镇建设。用城市眼光建设县城，完善规划布局，有序推进旧城改造与新区开发，抓好进出城通道和重要节点建设。充分发挥5个县域次中心的示范带动作用，加强福银高速公路互通口所在乡镇和边贸重点乡镇建设，抓好26个省级村镇住宅小区试点，积极稳妥实施撤乡并镇、迁村并点。

　　（七）繁荣各项事业，促进协调发展

　　加强科技、人才和教育。健全生产力促进中心、中小科技企业孵化器等科技服务体系，推动企业建立研发机构或技术中心，组织30项科技项目攻关，抓好50项产学研合作项目，推进高新技术企业和技术创新试点示范企业、制造业信息化试点示范企业建设。加大科普投入，保护知识产权，繁荣社会科学。加强各类人才培养，积极引进智力，发挥领军人物核心作用。巩固提高基础教育，推进“双高普九”，优化学校布局，实施第二期第三批中小学危房改造项目115个，加强教师队伍建设，深化初中课改和高中新课程改革。培育和扩大优质高中，扶持薄弱学校建设。建设一批高校基础设施项目，增创办学特色，提高办学水平。发展职业教育，扩大办学规模，促进中等职业教育和普通高中协调发展。支持办好民办学校。重视发展老年教育，健全终身教育体系。

　　推进社会事业协调发展。加强突发公共卫生事件应急体系和卫生监督体系建设，抓好重点传染病防控，做强重点医疗机构，发展规模民营医院，开展“看病难、看病贵”综合治理，加强妇幼卫生保健，开展爱国卫生运动，巩固国家卫生城成果。打造文化精品，抓好文物保护、利用，扩大万寿岩旧石器时代遗址等品牌影响，完善基层文化设施，活跃群众文化，加强文化市场管理。推动群众体育与竞技体育发展，力争在第十三届省运会上取得好成绩。加快广播电视网络化、数字化建设，提高广电节目制作质量。创新社会事业项目建设机制，形成多元化的发展格局。

　　促进人与自然和谐发展。落实计划生育工作机制和领导责任制，深化优质服务先进县创建活动，解决好出生人口性别比偏高、流动人口管理和服务难度大、社会抚养费征收不到位等问题。加强耕地保护，加快实施土地开发整理项目，确保耕地占补平衡。抓好水资源开发与管理，提高矿山集约开采和综合利用水平。开展地质灾害防治工作，加强水土流失治理。深入开展闽江流域三明辖区水环境综合整治，加大工业污染治理力度，开展汽车尾气、饮食业油烟等污染治理，抓好规模化养殖场污染整治，保护饮用水源，推进生态功能区建设，力争工业废水排放达标率93.5%%以上，市区空气质量良好天数达到75%以上。

　　（八）落实为民利民，构筑和谐三明

　　加强就业与社会保障。落实国务院新一轮就业再就业政策，立足发展扩大就业，争取全年城镇新增就业1.98万人，城镇下岗失业人员再就业0.95万人。切实解决拖欠农民工工资和建设工程款问题，加快建立城乡一体化就业工作机制。健全社会保障体系，完善城乡居民最低生活保障制度，探索解决困难企业退休人员参加医保问题，提高非公有制企业社会保险参保率。继续加强社会福利、社会救济、优抚安置和社会互助工作，发展慈善事业。

　　办好惠及群众的实事。集中财力抓好为民办实事项目。扶持老区加快发展，推进老区小康和科技示范基地建设，继续帮助解决“行路难”等生产生活问题。着力加强32个省级扶贫重点村和36个少数民族村建设，继续实施“造福工程”。实行县级以上医院挂钩帮扶乡镇卫生院，改造提升20所乡镇卫生院。扩大新型农村合作医疗试点和城乡医疗救助试点，对重点优抚对象和革命“五老”人员实施医疗救助。发展妇女、儿童和老龄事业，建设老年公寓、儿童乐园等项目。对农村义务教育阶段学生全部免除学杂费，继续实行农村低保家庭子女接受义务教育“两免一补”政策，进一步完善高校、中等职业学校家庭经济困难学生助学体系。继续推行农村部分计划生育家庭奖励扶助制度，深入开展“幸福工程—救助贫困母亲行动”。

　　营造和谐的社会环境。加强公民道德和未成年人思想道德建设，深入开展群众性精神文明创建活动，推进和谐社区、和谐村镇建设。建立、健全信用监管和失信惩戒机制。加强社会治安综合治理，减少青少年犯罪，严厉打击刑事犯罪和黄、赌、毒活动，防范和处理邪教组织非法活动，推进平安三明建设。加强普法宣传，畅通社情民意反映渠道，有效化解矛盾纠纷，落实突发公共事件应急预案，有序应对各类突发事件和风险。加强市场监管，保障食品、药品安全，保持农资价格平稳。落实安全生产责任制，严格安全生产执法，防范重特大事故发生。健全村（居）民自治制度，做好村（居）委会换届选举工作。深入开展“双拥”工作，推动国防动员、人民防空和民兵预备役建设。抓好民族、宗教、档案、地方志、气象、水文、库区移民、防震减灾等工作，保障残疾人合法权益。

　　（九）转变政府职能，提高行政效率

　　突出服务发展。我们要以胡锦涛总书记在福建视察工作的重要讲话精神为动力，增强加快发展的激情，保持加快发展的冲劲，集聚加快发展的气势，以奋发有为的精神状态促进三明更快更好发展。要主动融入大局，着眼海峡西岸，在更广的区域找站位，在更大的空间谋发展，把三明的企业、三明的品牌、三明的项目推向全国。要拓宽发展思路，勇于创造，善于争取，在已经领先的领域担当龙头，在可以领先的领域敢于争先，在能够突破的领域大胆突破。要提高服务效率，审批事项再减少，审批权限再下放，审批环节再精简，创新行政管理，推行电子政务，加强效能建设，开展绩效评估，转变政府职能。

　　强化工作落实。抓落实必须明确责任，责任不清则无人负责，无人负责就无人干事。我们要增强责任意识，提高运作能力，对部署的工作、作出的决策，能够扛得起、抓得住、干得好。抓落实必须强化项目带动，通过项目竞赛等载体，把项目带动拓展到经济社会发展的各领域，延伸到政府工作的全过程，使项目成为工作的着力点、发展的突破点、财税的增收点。抓落实必须攻坚克难，变困难为动力，化风险为机遇，善于消除经济运行中的不利因素，有效破解制约发展的深层次矛盾与问题。抓落实必须体现为民，有效落实惠民政策，有效服务基层，多体谅基层，多支持基层，让发展的主体活跃起来，发展的合力集聚起来，发展的氛围浓厚起来。

　　坚持依法行政。自觉把依法行政作为政府工作的基本准则，善于运用法律手段处理各种矛盾和问题，规范行政执法，严格实行执法责任制和执法过错责任追究制。认真执行人大及其常委会决议、决定，办好市人大代表建议、意见和市政协提案，重要工作主动与政协协商。拓宽各民主党派、工商联、无党派人士参政议政、民主监督的途径。加强廉政建设，建立健全教育、制度、监督并重的惩治和预防腐败体系，落实廉洁自律各项规定，推进政务公开，坚决纠正损害群众利益的不正之风，努力建设推动工作、为民负责、公正廉洁的公务员队伍。(摘要)
